# Burden and determinants of MDR-TB among prisoners in sub-Saharan Africa: Systematic review and meta-analysis protocol

**DOI:** 10.4102/jphia.v16i4.1364

**Published:** 2025-08-30

**Authors:** Doris Y. Sakala, Jacques M. Tamuzi, Constance S. Shumba, Peter S. Nyasulu

**Affiliations:** 1 Division of Epidemiology and Biostatistics, Faculty of Medicine & Health Sciences, Stellenbosch University, Cape Town, South Africa; 2 Department of Epidemiology and Social Sciences, Division of Epidemiology and Social Sciences, Institute for Health and Equity, Medical College of Wisconsin Milwaukee, Wisconsin, United States of America; 3 Division of Epidemiology and Biostatistics, School of Public Health, Faculty of Health Sciences, University of the Witwatersrand, Johannesburg, South Africa

**Keywords:** multi-drug resistance tuberculosis, sub-Saharan Africa, extensively drug resistance TB, correctional facilities, prisons

## Abstract

**Background:**

Tuberculosis (TB) is one of the leading causes of death globally because of a single infectious pathogen. The rise in prevalence of multi-drug-resistant tuberculosis (MDR-TB) puts an increased burden on the health system in terms of cost and longer treatment duration. People living in correctional facilities are more likely to develop TB and have poor TB treatment outcomes than the general population, making them a vulnerable group to develop MDR-TB. However, the burden of MDR-TB and associated treatment outcomes among prisoners in sub-Saharan Africa (SSA) is poorly documented.

**Aim:**

The study aims to investigate the burden and associated factors of MDR-TB treatment among prisoners in SSA.

**Setting:**

The review will include studies of MDR-TB done in prisons and detention centers involving prisoners and inmates in sub Saharan Africa.

**Methods:**

Following the Preferred Reporting Items for Systematic Reviews and Meta-Analyses (PROSPERO), we will conduct a systematic review and meta-analysis. We will review studies examining MDR-TB patient treatment outcomes among prisoners reported in published literature in SSA from 2000 to 31 December 2024. A search on studies reporting MDR-TB treatment outcomes from the databases such as ‘Medline, Embase, CINAHL (EBSCOhost), Scopus and Web of Science’ will be conducted. We will analyse continuous outcomes as mean differences for studies using the same scales with standard deviation reported and binary outcome data as odds ratios or risk ratios, all presented with their 95% confidence intervals. Additionally, the pooled proportions will be used to determine the prevalence or incidence of specific MDR-TB treatment outcomes. Heterogeneity will be assessed using the *I*^2^ statistic, and where significant heterogeneity is detected, a random-effects model meta-analysis will be performed; otherwise, a fixed-effect model meta-analysis will be carried out. Risk factors will be determined using the meta-regression analysis technique.

**Results:**

After analysis of pooled data, prevalence of MDT-TB in prisons will be presented as proportions. Meta-analysis outcome will be presented as forest plots, showing odd ratios and co-responding 95% confidence intervals. Narrative synthesis of included studies will be presented in a table format.

**Conclusion:**

This proposed systematic review and meta-analysis will help consolidate evidence to support the development of public health guidelines to enhance the reduction of MDT-TB factors among prisoners in the SSA region.

**Contribution:**

This review will provide evidence to support guideline development on screening, diagnosis, and clinical management of MDR-TB patients in prisons.

## Introduction

Tuberculosis (TB) continues to be a significant global public health issue, responsible for approximately 10.6 million people developing TB worldwide in 2022, of which 1.3 million died.^1,2^ Multi-drug-resistant tuberculosis (MDR-TB) accounts for 20% of global TB deaths. Research studies have reported the global prevalence of MDR-TB to be at 3.2% of all new TB cases and 16% of previously treated cases in 2023.^1^ Several factors have been reported to be responsible for exacerbating the emergence and spread of drug-resistant TB, such as transmission within high-risk settings and disproportionately affecting vulnerable populations, including prisoners, human immunodeficiency virus (HIV) patients, migrants, unhoused people and drug users. More recent studies have also noted that previous diagnosis and onset of TB treatment are associated with poor treatment success rates among 45%–50%^1,2,3,4,5,6,7,8^ of prisoners and have potentially been identified as a source of MDR-TB and extensively drug-resistant TB (XDR-TB) infections. In addition, a study quantified the effect of incarceration on the transmission of MDR-TB and further highlighted the role of prisons in the epidemic of MDR-TB.^7^

Global incidence estimates reported by WHO in 2019 showed that 125 105 prisoners worldwide developed TB, representing about 1% of the global incidence.^1^ Disturbingly, about half of these cases went unidentified, underscoring the critical need for enhanced TB control measures in correctional facilities to improve associated treatment outcomes.^5,6,7,8,9^ International and national TB guidelines, including those for HIV and TB coinfection and programmes for high-risk groups, have directed their attention on case detection while regularly updating preventive measures. Despite these efforts, relatively little effort and attention are paid to populations in correctional facilities, thus affecting the development and implementation of effective policies for these individuals.^4,10^

Evidence suggests that the risk of developing TB in prison is 6–30 times higher than in the general population but 200 times higher in sub-Saharan Africa (SSA), particularly in overcrowded prisons, such as in the Democratic Republic of the Congo (DRC), Zambia and Ethiopia.^2^ On the other hand, MDR-TB treatment in SSA faces several significant challenges that hinder effective patient treatment outcomes.^2,4^

These include delayed diagnosis because of limited access to advanced diagnostic tools, such as GeneXpert and culture-based drug susceptibility testing (DST), which contribute to significant delays in treatment initiation.^11,12^ The failure to diagnose and effectively treat MDR-TB patients perpetuates ongoing MDR-TB transmission in communities and has been linked to increased MDR-TB burden in the region.^13^ Although detection of MDR and treatment enrolment have improved globally over the past decades, the global treatment success outcome rate (defined as cured or treatment completed) for MDR-TB was found to be sub-optimal at only 59% in 2020.^8,13^ Treatment adherence is another critical issue, as the lengthy and often toxic MDR-TB regimens lead to poor adherence among patients, increasing the risk of resistance and treatment failure.^14,15,16^ Low adherence increases the risk of poor treatment outcomes, including treatment failure, relapse and development or amplification of drug resistance.^17^ In SSA, loss to follow-up rates range from 11.3% to 29.6%, which exacerbates the emergence of drug-resistant strains and prolonged infectiousness.^16^ Furthermore, the high rates of defaulting on treatment are often linked to inadequate healthcare support, stigma associated with TB and insufficient patient education regarding treatment importance.^16,18^

The burden of TB in prisons in SSA is alarmingly high and has been compounded by factors such as overcrowding, high rates of HIV coinfection, inadequate healthcare infrastructure and poverty.^10,11,12,19^ The prevalence of all forms of TB is 3.5 times higher. Bacteriologically confirmed TB is 1.7 times higher, and MDR-TB is 3.5 times higher than in the general population.^13^ Notwithstanding the recognised high burden of TB in correctional facilities, there is a lack of data on TB treatment outcomes and the factors influencing these outcomes among prisoners.

Understanding MDR-TB treatment outcomes among prisoners in SSA is critical. In countries with a high TB burden, coupled with the augmented susceptibility of prisoners to TB, focused research is required to enlighten effective MDR-TB control approaches. Also, MDR-TB in correctional facilities can have substantial public health consequences, as prisoners may add to the spread of TB in the general community because of contact with friends and families during visits. Further, data on treatment outcomes can direct the enactment of customised interventions to improve MDR-TB control in SSA correctional facilities. Lastly, the results can help create policies and guide resource allocation to improve MDR-TB treatment and control in prisons. Thus, this study’s objective is to conduct a comprehensive systematic review and meta-analysis to determine factors associated with multi-drug-resistant tuberculosis treatment outcomes among prisoners in SSA.

## Research methods and design

This protocol has been prepared according to a statement recommendation made by the Preferred Reporting Items for Systematic Review and Meta-Analysis Protocols (PRISMA-P) in 2015. The enhanced six-stage methodological framework for systematic review and meta-analysis will be used.^20^ We will strictly follow the PRISMA flow diagram during the process of study selection.

### Objective

The objective of this systematic review and meta-analysis is to determine the burden and the factors that are associated with MDR-TB treatment outcomes among prisoners in SSA.

### Criteria for studies included in the systematic review

Any study drawn from observational studies such as cohort, case-control and cross-sectional studies that analyse the incidence, prevalence, mortality, completion of treatment, treatment failure, loss to follow-up and cured MDR-TB treatment outcomes among prisoners reported in SSA countries will be included. On the other hand, only studies that reported a well-defined sample or representative sub-sample of the source population will be eligible for inclusion. Though studies that assess intervention effectiveness will not be the focus of this systematic review, randomised controlled trials (RCTs) that reported baseline data to allow the calculation of the proportion of MDR-TB treatment outcomes will be eligible.

### Study participants

We will include prisoners incarcerated in SSA countries who have been diagnosed with MDR-TB. The criteria for the identification of the prisoners will be explicitly stated. Any participant whose MDR-TB treatment outcome could not be confirmed will be excluded from the study. Prisoners incarcerated in SSA will be compared according to MDR-TB risk factors, including HIV, diabetes, alcohol, smoking and nutritional status.

### Exposures

Prisons are considered as a risk factor for MDR-TB as they are often high-risk environments for TB transmission because of severe overcrowding, poor nutrition, poor ventilation and limited access to often comprehensive health care.

### Study outcomes

- **Mortality:** A prisoner with proven MDR-TB dies for any reason before or during treatment.
- **Lost to follow-up:** A prisoner who did not start treatment or whose treatment was interrupted for at least 2 months in a row.
- **Treatment failure:** A prisoner who is sputum smear or culture positive at least 5 months into treatment.
- **Treatment completion:** A prisoner who completed treatment without evidence of failure but without sputum smear or culture results.
- **Cured:** A prisoner with bacteriologically confirmed TB who is smear- or culture-negative at the end of treatment and at least once before.
- **Transfer out:** A transfer outpatient is a patient who has been transferred out at any time during treatment to continue treatment at another facility.
- **Incidence:** All new cases reported.

### Data extraction and management

A data extraction sheet will be developed, pretested and used for data collection. At least two reviewers will extract information on the characteristics of the studies, including the year the study was conducted, the year it was published, the country where the study was conducted, the study design and the number of participants involved (sample size). Sociodemographic characteristics of the participants such as age, level of education, occupation, setting and marital status as well as some important clinical conditions will also be extracted. The data on the seven outcomes will also be abstracted.

The extracted data will be cleaned up and managed with Microsoft Excel, where all transformations and conversions needed for the analysis will be done. Any discrepancies or disagreements in the data extracted will be resolved through discussion between the review authors.

### The eligibility criteria

A search grid will be created using the Population, Intervention, Comparator and Outcome (PICO)^21,22^ model recommended by Joanna Briggs Institute (JBI). P refers to prisoners in SSA, I is the exposure (E), which refers to conditions exposed to MDR-TB in prisons, C refers to comparing prisoners with MDR-TB with HIV and those without HIV and O refers to the outcomes, including mortality, treatment failure, treatment completion, cured or transfer out. This systematic review and meta-analysis will include all observational study designs (cross-sectional, case-control and cohort studies) with MDR-TB patients receiving treatment (bacteriologically, clinically confirmed MDR-TB and new or recurrent MDR-TB). In addition, studies published in the English language as well as other languages and reporting TB treatment outcomes in relation to HIV coinfection will be considered for the final analysis. No restrictions will be applied in terms of publication status, study and publication year, study setting or sampling technique. However, papers that will not be fully accessible at the time of our search process will be excluded. We will contact the authors via email at least 2–3 times, after which the papers will be excluded.

## Identifying relevant studies

### Electronic databases

We will retrieve all studies (published and unpublished) and assess their eligibility for inclusion. The following databases will be searched: PubMed, Embase, PsycINFO, CINAHL, LILACS, Google Scholar, Scopus and Web of Science from 2000 to 31 December 2024, without language restriction. The key search terms and concepts include ‘MDR’, ‘TB treatment outcomes’ and ‘SSA’ (see Table 1 for the search strategy developed for PubMed, which will be adapted for the other databases). Grey sources, such as conference proceedings, dissertations, preprint repositories, the World Bank Open Knowledge Repository, World Health Organization (WHO), Program for Appropriate Technology in Health (PATH) and United Nations Children’s Fund (UNICEF) databases, will be checked for more studies. The reference lists of relevant articles will be reviewed, and contacts with field experts will be made for information about any study missed by the researchers. D.Y.S. and J.L.T. will separately evaluate the abstracts of the necessary complete texts to determine whether they are eligible. These reviewers will do an additional independent examination of the full-text papers for the titles that have been selected. Disagreements between these reviewers will be resolved through conversations with the third reviewer (P.S.N., C.S.S.).

**TABLE 1:** Search strategy for PubMed (to be tailored to other databases).

Search	Search strategy	Result
#1	Treatment outcome [Title/Abstract] OR Cured [Title/Abstract] OR mortality [Title/Abstract] OR Lost to Follow-up [Title/Abstract] OR Completed treatment [Title/Abstract] OR treatment Failure [Title/Abstract] AND Prisoners	-
#2	(((((((((((((cured[All Fields] OR ((“therapy”[Subheading] OR “therapy”[All Fields] OR “therapeutics”[MeSH Terms] OR “therapeutics”[All Fields]) AND cured[All Fields])) OR (“treatment failure”[MeSH Terms] OR (“treatment”[All Fields] AND “failure”[All Fields]) OR “treatment failure”[All Fields])) OR (“therapy”[Subheading] OR “therapy”[All Fields] OR “therapeutics”[MeSH Terms] OR “therapeutics”[All Fields])) OR (“lost to follow-up”[MeSH Terms] OR (“lost”[All Fields] AND “follow-up”[All Fields]) OR “lost to follow-up”[All Fields] OR (“lost”[All Fields] AND “follow”[All Fields] AND “up”[All Fields]) OR “lost to follow-up”[All Fields])) OR Transferred[All Fields]) OR incomplete[All Fields]) OR (incomplete[All Fields] AND (“Microb Drug Resist”[Journal] OR “mdr”[All Fields]) AND (“therapy”[Subheading] OR “therapy”[All Fields] OR “treatment”[All Fields] OR “therapeutics”[MeSH Terms] OR “therapeutics”[All Fields]))) OR (“mortality”[Subheading] OR “mortality”[All Fields] OR “mortality”[MeSH Terms])) OR (“death”[MeSH Terms] OR “death”[All Fields])) OR complete[All Fields]) OR (complete[All Fields] AND (“Microb Drug Resist”[Journal] OR “mdr”[All Fields]) AND (“therapy”[Subheading] OR “therapy”[All Fields] OR “treatment”[All Fields] OR “therapeutics”[MeSH Terms] OR “therapeutics”[All Fields]))) OR (successful[All Fields] AND (“treatment outcome”[MeSH Terms] OR (“treatment”[All Fields] AND “outcome”[All Fields]) OR “treatment outcome”[All Fields]))) OR (successful[All Fields] AND (“treatment outcome”[MeSH Terms] OR (“treatment”[All Fields] AND “outcome”[All Fields]) OR “treatment outcome”[All Fields]))) AND (“prisons”[MeSH Terms] OR “prisons”[All Fields])	-
#3	#1 AND #2	-
#4	(((((((((((((((((((((((((((((((((((((((((((((((((“Democratic Republic of Congo”[Title/Abstract]) OR (“Republic of Congo”[Title/Abstract])) OR (“Central African Republic”[Title/Abstract])) OR (Rwanda[Title/Abstract])) OR (Burundi[Title/Abstract])) OR (Sudan[Title/Abstract])) OR (Kenya[Title/Abstract])) OR (Tanzania[Title/Abstract])) OR (Uganda[Title/Abstract])) OR (Djibouti[Title/Abstract])) OR (Eritrea[Title/Abstract])) OR (Ethiopia[Title/Abstract])) OR (Somalia[Title/Abstract])) OR (Angola[Title/Abstract])) OR (Botswana[Title/Abstract])) OR (Lesotho[Title/Abstract])) OR (Malawi[Title/Abstract])) OR (Mozambique[Title/Abstract])) OR (Namibia[Title/Abstract])) OR (“South Africa”[Title/Abstract])) OR (Swaziland[Title/Abstract])) OR (Zambia[Title/Abstract])) OR (Zimbabwe[Title/Abstract])) OR (Benin[Title/Abstract])) OR (“Burkina Faso”[Title/Abstract])) OR (Cameroon[Title/Abstract])) OR (Chad[Title/Abstract])) OR (“Côte d’Ivoire”[Title/Abstract])) OR (“Equatorial Guinea”[Title/Abstract])) OR (Gabon[Title/Abstract])) OR (Gambia[Title/Abstract])) OR (Ghana[Title/Abstract])) OR (Guinea[Title/Abstract])) OR (Guinea-Bissau[Title/Abstract])) OR (Liberia[Title/Abstract])) OR (Mali[Title/Abstract])) OR (Mauritania[Title/Abstract])) OR (Niger[Title/Abstract])) OR (Nigeria[Title/Abstract])) OR (Senegal[Title/Abstract])) OR (“Sierra Leone”[Title/Abstract])) OR (Togo[Title/Abstract])) OR (“Cape Verde”[Title/Abstract])) OR (Comoros[Title/Abstract])) OR (Madagascar[Title/Abstract])) OR (Mauritius[Title/Abstract])) OR (“São Tomé and principle”[Title/Abstract])) OR (Seychelles[Title/Abstract])) OR (“Sub-Saharan Africa”[Title/Abstract])) OR (SSA[Title/Abstract])	-
#5	#3 AND #4	-

### Ethical considerations

This article followed all ethical standards for research without direct contact with human or animal subjects. This study will use publicly available data, and thus ethics approval is exempted. The protocol is registered on the Prospective Register of Systematic Reviews (PROSPERO) 2025, registration number CRD420251000077, in February 2025. The findings of the systematic review will be disseminated on various platforms, including peer-reviewed journals, digital media platforms such as webinars as well as seminars and conferences.

## Results

### Search results and selecting studies

**FIGURE 1:**
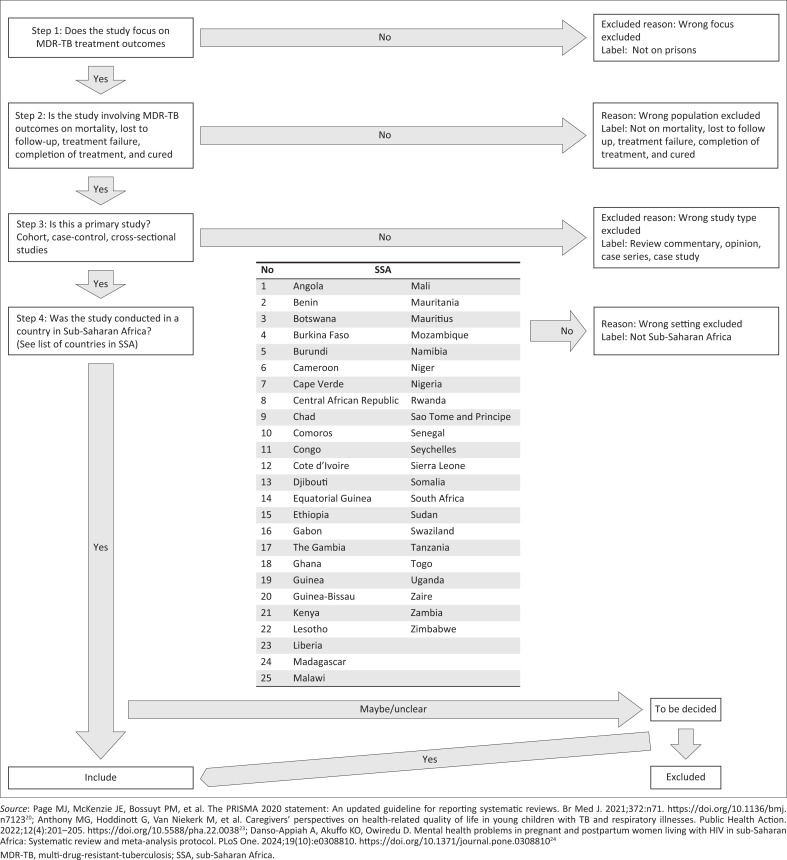
Diagram showing inclusion and exclusion criteria.

The studies will be screened and selected by two independent reviewers. Potential studies will be retrieved and uploaded into EndNote version X9, where duplicates will be removed. The duplicated articles will be exported to Excel, where study selection will be conducted using some preset selection criteria as shown in Figure 1.

**FIGURE 2:**
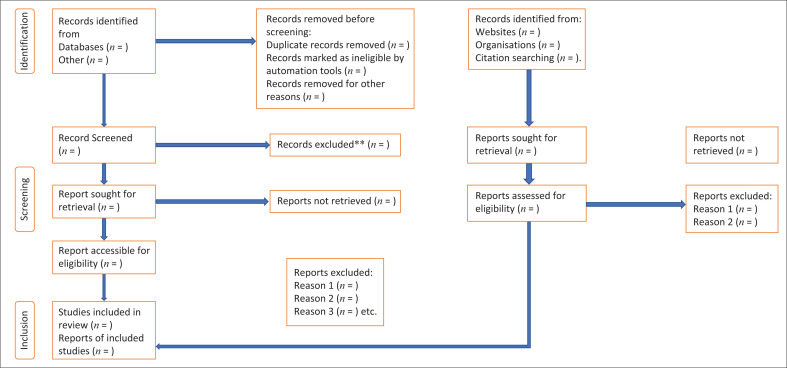
Flowchart diagram of MDR-TB prisoners in SSA.

Firstly, titles and abstracts will be screened to remove completely irrelevant studies. Secondly, the full text of articles that potentially meet the inclusion criteria will be sought and screened for inclusion. At this stage, any study that did not meet the inclusion criteria will be excluded, and the reason for exclusion will be provided. The flow of studies through the selection process will be presented using the PRISMA flow diagram shown in Figure 2. Any discrepancies or disagreements will be resolved through discussion. When needed, a third team member (P.S.N. or C.S.S.) will intervene to mediate conflicts.

### Quality assessment (risk of bias)

At minimum, two reviewers will independently evaluate the quality of the included studies for risk of bias using the available standard tool known as the Cochrane tool for non-randomised studies of exposure (Robbins-E). This tool consists of a series of questions across seven risks of bias domains, including (1) confounding, (2) study participants’ selection, (3) exposure measurement, (4) post-exposure intervention, (5) missing data, (6) outcome measurement and (7) how reported results were selected. Each question in the domain has ‘Yes’, ‘Probably Yes’, ‘Probably No’, ‘No’ and ‘No Information’ responses. The judgement on the risk of bias will be reported as ‘Low’ for a domain with little or no concern about bias; ‘Some concerns’ means there are issues about bias in a specific domain but with no certainty of an important risk of bias, ‘High risk’ for domains with some important bias concerns and ‘Very high risk of bias’ for studies with suspected serious bias. The risk of bias in prevalence studies will be assessed using the Hoy et al.^5,24^ quality assessment tool. This tool consists of 10 items that address four risk of bias domains: (1) representativeness of the study sample to the source population, (2) representativeness of the sampling frame to the target population, (3) reliability of the sampling, (4) likelihood of minimisation of nonresponse bias, (5) data collected directly from the subjects (as opposed to a proxy, (6) acceptable case definition, (7) validity and reliability of study instrument that measured the parameter of interest, (8) mode of data collection used, (9) appropriateness of the prevalence period for the parameter of interest and (10) appropriateness of the numerator(s) and denominator(s) for the parameter of interest.

The domains of the proposed tool assess research constructs ranging from internal to external validity. The first four domains assess external validity and domains 5–10 assess internal validity. The risk of bias in each domain will be judged as ‘Low risk of bias’, ‘Moderate risk of bias’, ‘Serious risk of bias’, ‘Critical risk of bias’ and ‘No information’, as guided in the Cochrane manual. The internal validity domains will be the basis for scoring the overall risk of bias in each of the included studies as ‘low’ or ‘high’ risk of bias. Disagreements between the two reviewers (D.Y.S. and J.L.T.) will be resolved through discussion between the review authors and the senior researchers (P.S.N. and C.S.S.).

### Statistical analysis

We will use R software version 4.4 for data analysis. Binary outcome data will be analysed as odds ratio (OR) or risk ratio (RR) and continuous data as mean difference (MD). In case the binary data lack comparisons, the MDR-TB prevalence among prisoners will be assessed.

All estimates will be reported with 95% confidence intervals (CIs). Meta-analysis will be conducted when at least two similar studies are available to determine the pooled OR, RR or MD of the individual studies. The analyses will be presented graphically as forest plots. Heterogeneity arising from included study designs and participant characteristics that are likely to lead to differences in outcomes between studies will be assessed quantitatively using the *I*-squared (*I*^2^) statistics. If there is no significant heterogeneity (*I*^2^ < 50%), we will utilise fixed-effects models to pool results. When significant heterogeneity is present (*I*^2^ ≥ 50%), we will employ random-effects models. The ‘DerSimonian and Laird method’ will be used to assess the overall effect size when the studies included in the meta-analysis are heterogeneous. In case the heterogeneity between included studies is not significant, a fixed-effect model will be appropriate to help obtain pooled estimates for MDR-TB treatment outcomes among prisoners, such as mortality, completion of treatment, treatment failure, lost to follow-up and cured. Subgroup analysis will be performed to investigate the differences between prisons in Southern, Central, Western and Eastern SSA if there is enough data available. If a meta-analysis includes ten or more papers, a funnel plot will be created to evaluate publication bias. When appropriate, statistical tests for funnel plot asymmetry (Egger test, Begg test and Harbord test) will be conducted. To investigate the factors associated with MDR-TB treatment outcomes (including age, gender, MDR-TB regimens and drugs’ side effects), a meta-regression, including potential factors with MDR-TB (age, gender, smoking, nutritional status, etc.), will be carried out. Lastly, the leave-out-one sensitivity analysis will be performed on outlier studies to test the robustness of the pooled estimates.

### Assessing the certainty of evidence using Grading of Recommendations Assessment, Development and Evaluation

Certainty assessment of evidence will be done using the Grading of Recommendations Assessment, Development and Evaluation (GRADE).^25,26^ This approach is important for assessing the overall quality or certainty of the evidence (how certain the authors are that the effect estimate represents the true effect) on the key outcomes provided by the included individual studies. As highlighted, at minimum, two reviewers will help assess the certainty of evidence (quality of evidence or the confidence in the effect estimates) covering the following five domains: risk of bias, inconsistency, indirectness, imprecision or publication bias. The output of the certainty of evidence evaluation will be graded as ‘Low risk of bias’, ‘Moderate risk of bias’, ‘Serious risk of bias’, ‘Critical risk of bias’ and ‘No information’. Publication bias will be determined using both visual and statistical techniques. Inspection using funnel plots and the Egger test will formally help to determine the small study effect. Further, substantial asymmetry or significant test of the Egger test results (*p* < 0.05) will result in the modification of the variation of evidence in the GRADE framework.^27^ Several parameters will be used in the interpretation of heterogeneity, number of studies and other potential causes of asymmetry. An overall rating of the certainty of the evidence for each outcome, together with the study types, the number of studies and participants and the relative and absolute effects for each outcome, will be presented in a summary of findings table. Publication bias will be summarised using a funnel plot and the Egger regression test technique.

## Discussion

This systematic review and meta-analysis protocol is anchored in the urgent need to address the recognised notable gap in the literature regarding MDR-TB burden among prisoners and also the need to identify risk factors driving this burden in SSA. Despite the increasing recent evidence showing the increased burden and selected risk factors such as demographics, comprehensive evidence on the burden, risk factors and outcomes related to MDR-TB among the prison population remains deficient.^28,29,30,31,32^ This protocol aims to address a fundamental public health problem by providing a detailed plan for collating available peer-reviewed unbiased evidence regarding MDR-TB health problems among prisoners in SSA.

Human immunodeficiency virus-resistant tuberculosis is a serious public health issue that affects patient treatment outcomes, especially prisoners’ well-being, in profound ways. The relationship between demographic factors and treatment outcomes has been the subject of numerous studies, yielding different outcomes for various groups and regions, especially in SSA.^28,29,33,34,35^ This systematic review is the very first attempt to compile evidence, in a comprehensive manner, on MDR-TB treatment outcomes, including successful and unsuccessful outcomes among prisoners in SSA, to estimate the magnitude and risk factors influencing health outcomes of prisoners in SSA. This review will be useful for shaping policies on prevention and management of MDR-TB in prisons. This review employs rigorous methods to address both external and internal validity concerns. The external validity employs the PICOS to concisely delineate the attributes of the participants, interventions, outcomes and study designs, whereas the internal validity addresses mainly the risk of bias (quality) domains. This review gathers evidence through a very thorough search strategy that aims to locate all potentially relevant studies from a variety of study designs, including cohort, case-control and cross-sectional studies. It does this by using transparent and robust methods. It is anticipated that this review will provide policymakers, health providers and other stakeholders with evidence-based information and inspire countries to adopt innovative interventions to limit adverse outcomes among prisoners.

### Study limitations

We expect that studies on MDR-TB in prisons might be reported, thereby resulting in a possible publication bias. This is likely to happen, as most prison authorities regard resistance negatively because of its potential to reflect the poor institutional control efforts. The review may include research from various settings with distinct healthcare systems and cultural norms, which further limits the generalisability of the findings because of geographic and socioeconomic disparities in the treatment outcomes of MDR-TB in prisons. Comprehensive analysis may also be limited by incomplete reporting of relevant outcomes.

### Potential implications of the anticipated review findings

The strength of the anticipated results of this review will be the pooling of findings from studies using a comprehensive database search, describing the emerging and ongoing burden prevalence and risk to MDR-TB treatment outcomes for prisoners. This would be useful for shaping policies, clinical practice, patient management and research in SSA. This review will highlight critical evidence that will draw attention to the need for stronger MDR-TB healthcare services by highlighting the increased risks of mortality and transmission, treatment failure, treatment completion, lost to follow-up and cured outcomes among prisoners. The results could also point to differences in health outcomes, according to several risk factors such as socioeconomic and regional characteristics, highlighting the need for equitable resource allocation and focused public health interventions for MDR-TB patients in resource limited settings. These findings may help shape health policies that support better access to MDR-TB treatment, active MDR-TB surveillance and education and discourage practices that may lead to poor MDR-TB treatment outcomes. This review may also point out gaps in the current body of knowledge about MDR-TB among prisoners and its associated treatment outcomes, which could address understudied topics and, in the end, improve the treatment outcomes. These results will be very important in achieving the SDG3 goal and target 3.3 of attaining a 90% reduction in the number of TB deaths and an 80% reduction in the TB incidence rate (new cases per 100 000 population per year) by 2020 compared with the levels in 2015.

### Strength of the study

Despite the noted limitation of this study, we feel that the proposed protocol provides a reliable methodology to collate and refine pragmatic evidence at the highest level using reliable, reproducible, unambiguous techniques to answer overarching review questions while adhering to best practices known for critically appraising the research evidence.^24,35^ The proposed protocol lays down a clear and robust process, which will involve the development of a comprehensive search strategy that will be searched up in multiple databases, both medical and non-medical, in a bid to retrieve all potentially relevant studies.
